# A Flexible Annular Sectorial Sensor for Detecting Contact Position Based on Constant Electric Field

**DOI:** 10.3390/mi9060309

**Published:** 2018-06-19

**Authors:** Haibin Wu, Haomiao Wang, Jianfeng Huang, Youzhi Zhang, Jinhua Ye

**Affiliations:** School of Mechanical Engineering and Automation, Fuzhou University, Fuzhou 350116, China; wuhb@fzu.edu.cn (H.W.); wanghaomiao.cn@gmail.com (H.W.); huangjianfeng654@gmail.com (J.H.); zhangyouzhi606@gmail.com (Y.Z.)

**Keywords:** flexible sensor, constant electric field, detection of contact position, electrical potential, finite element analysis

## Abstract

To achieve tactile detection on the irregular surface of a robot link, a flexible annular sectorial sensor with a five-layer structure was proposed that could be wrapped on the surface of a truncated cone-shaped link. The sensor was designed for the detection of a contact position when robots collide with other objects during movement. The sensor obtains the coordinates of the contact position by exerting a constant electric field on the upper and lower conductive layers. The mathematical model linking the coordinates of the contact position and the corresponding electric potential on the conductive layer was established, based on the uniqueness of the electric field. The design of the sensor was simulated using COMSOL software, and the detection error of the contact position was discussed. A sensor sample was fabricated and wrapped on the mechanical arm. The results of the simulations and experiments indicated that the flexible sensor performed very well when wrapped on the robot link.

## 1. Introduction

Tactility is an important perceptive function for interaction between robots and various environments. For safety, control and communication, robots in unstructured environments should sense information in a similar manner to humans. Using machine vision to complete the interaction with environments has been frequently used. However, shadows may be present, and this leads to unreliable results [1]. Hence, developing a satisfactory tactile sensor is crucial for robot development [2,3]. Immediate feedback of external tactile information can fulfill the most basic requirements of safety. Tactile sensors provide information, e.g., contact pressure, contact position, surface texture and temperature, to guarantee safety in the process of human-robot interaction [4,5]. Tactile sensors can be categorized as ‘perception for action’ and ‘action for perception’ based on the task to be done. Perception for action mainly covers grasp control, contact point estimation and slip detection, whereas action for perception includes exploration, object recognition, hardness, etc. Furthermore, tactile sensors can be divided into intrinsic tactile sensing and extrinsic tactile sensing based on the location of the sensor. Intrinsic tactile sensing can be achieved by joint angle sensors or force/torque sensors. Extrinsic tactile sensing can be through distributed pressure/force/stress sensors or temperature sensors [6]. Tactile sensors can be divided according to their functional principle, into piezoresistive tactile sensors [7], piezoelectric tactile sensors [8], capacitive tactile sensors [9], magnetic tactile sensors [10], optical tactile sensors [11], etc. Contact position and force are the most important pieces of information for avoiding collisions in human-robot interaction, so there is a great deal of literature devoted to such functions. One of the basic requirements of tactile sensors is flexibility. In order to detect contact force at different positions, tactile sensors should be first wrapped around the robot. Robot structures are diverse in shape, so any tactile sensor with a large area must consider flexibility. Suen et al. [12] fabricated a 3 × 3 sensor array to mimic human skin, with high force sensitivity, high flexibility and able to measure temperature. The pressure and temperature in the contact area were detected by changes in resistance and thermal resistance of zinc oxide nanorods sandwiched between the top and bottom electrode layers. Ji et al. [13] presented an 8 × 8 tactile sensing array intended for use as a robot skin and based on a capacitive sensing mechanism. A microstructured polydimethylsiloxane was used to enhance the array’s sensitivity. The optimal sensitivity achieved 35.9%/N in a force range of 0–1 N. Yu et al. [14] proposed a new flexible piezoelectric tactile sensor array based on polyvinylidene fluoride film, for measuring the three-axis dynamic contact force distribution. The sensor array was arranged as a 3 × 2 matrix with 8-mm spacing between neighboring units. The above-mentioned tactile sensors are almost all structured as an array, and every patch of sensors requires a data acquisition module, which means a complex set of signaling circuits and a large amount of signal processing. In addition, detection is discontinuous and unsuitable for larger surface wrapping.

The outside surfaces of robots are normally irregular in shape. A kind of tactile sensor that resembles a textile with continuous detection is expected to wrap around the robot surface. Büscher et al. [15] introduced a novel fabric-based, flexible and stretchable tactile sensor, which can cover various shapes. The sensor detected pressure by the change of resistance in the piezoresistive fabric placed between two highly conductive materials. Pan et al. [16] designed a five-layered tactile sensor that could sense both the position and pressure of an external force, as well as carrying out continuous sensing. Two conductive fabric layers detect contact position; one partially conductive central layer detects contact force; and two insulating layers separate the outer layer and the central layer when there is no outside pressure present. In our previous work [17], we proposed a flexible tactile sensor based on the principle of a distributed planar electric field. The sensor had a non-array structure that could continuously detect the contact position. It could also cover the surface of a robot arm to guarantee safety. However, in our previous work and for other relevant continuous detection sensors, the shapes were almost all rectangular or circular. They are not suitable for wrapping over the irregular surface of a robot link as they cannot be wrapped on the robot link seamlessly. A comparison between the characteristics of sensors is shown in Table 1.

To solve the problem of wrapping sensors on the irregular surface of a robot link, this paper proposes a flexible annular sectorial sensor that can be wrapped on the surface of a truncated cone-shaped link and can perform continuous detection of contact position. The proposed sensor has a simple structure and few signal processing requirements. Compared with rectangular sensors, the annular sectorial sensor is more adaptable for joints and on a non-cylindrical link. This kind of sensor can be wrapped around the surface of an irregular link by changing the parameters of the annular sector.

In order to realize the function analyzed above, a structure with five-layers was designed. There are two main conductive layers that pass two independent constant electric fields through the structure. The mathematical models of the two electric fields were constructed using boundary conditions. Next, the coordinate of the contact position was calculated combined with a detection potential. This paper is organized as follows. Section 2 introduces the boundary value problem and constructs the mathematical model of the proposed sensor. Section 3 analyzes the precision of the sensor using simulations. Section 4 conducts an experiment to fabricate the sensor sample and to analyze the precision of the sensor sample. Section 5 reports the main results of the paper.

## 2. Principles and Methods

### 2.1. Uniqueness Theorem of Constant Electric Field

When a conductor is connected to the positive and negative poles of a direct current (DC) power supply, it creates an electric field around itself because of the potential difference of two electrodes. Free electrons move in a regular direction to form the current under the action of the electric field force, which includes an electric current field. The electric field that accompanies the current field is excited by all charges in the space. If we want to maintain a constant current distribution, the charge density everywhere should be maintained and should not change with time. In this way, the potential value of every point in the electric field is known and unchangeable.

### 2.2. The Establishment of a Sensor Coordinate System

**Figure 1:**
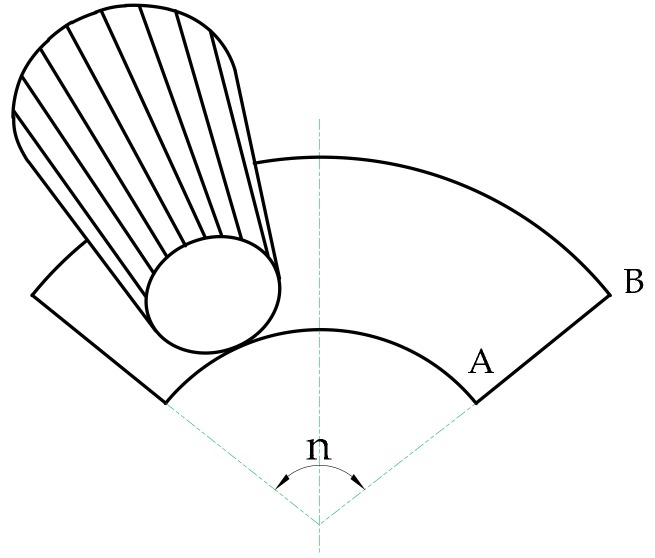
Unfolding the side of a truncated cone-shaped link.

According to geometry, the side of a truncated cone is an annular sector, as seen in Figure 1.

**Figure 2:**
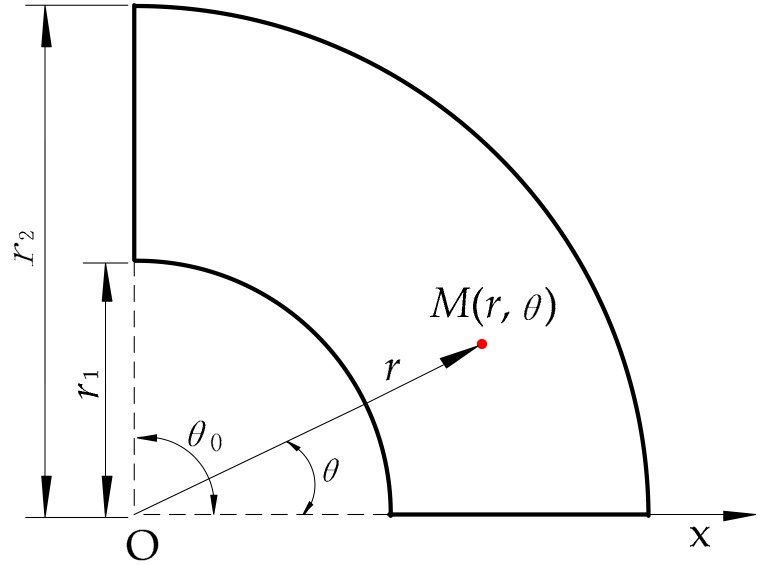
The establishment of a polar coordinate system on the annular sector.

The essence of wrapping a truncated cone-shaped link is to wrap the side of the truncated cone; hence, the shape of the proposed sensor is annular sectorial. Using the geometric properties of the annular sector, we established a polar coordinate system on the sensor, as shown in Figure 2.

The parameters *r*_1_, *r*_2_ and *θ*_0_ in Figure 2 are adjustable based on the dimension of the target link. *M*(*r*, *θ*) is the coordinate of the contact point under a polar coordinate system on the annular sectorial sensor, where *r* is the polar radius of *M* and *θ* is the polar angle of *M*. Thus, we obtain a model with parameters *r* and *θ* as coordinates of a contact point.

### 2.3. The Theoretical Model of a Constant Electric Field

We required constructing two constant electric fields in two independent dimensions and constructing two gradient functions of potential distribution to detect contact position. We could apply a potential difference to each of the two annular sectorial conductive layers in different ways in order to construct the two constant electric fields. The final formula of electric potential and the corresponding coordinate was deduced from a theoretical model for a constant electric field.

As in electromagnetics, a constant electric field is irrotational and without a source. It is constant in source-free regions. Therefore, the constant electric field satisfies the following basic equations:

(1) $$\{\begin{array}{l} \nabla \times E=0\\ \nabla \cdot J=0\\ J=\gamma E \end{array}$$

The electric field strength is equal to the negative of the potential gradient, i.e., E=−∇φ; where:

(2) $$\nabla =i_{x}\frac{\partial }{\partial x}+i_{y}\frac{\partial }{\partial y}+i_{z}\frac{\partial }{\partial z}$$

From the above basic equations,

(3) ∇⋅J=∇⋅γE=−γ∇⋅∇φ+E⋅∇γ=0

where γ is a constant in a homogeneous medium, and therefore, ∇γ=0 in Equation (3). We can obtain the Laplace equation of the potential φ in a constant current field.

(4) $$\nabla^{2}\varphi =0$$

In cylindrical coordinates, the Laplace equation is expressed as:

(5) $$\nabla^{2}\varphi =\frac{1}{\rho }\frac{\partial }{\partial \rho }\left(\rho \frac{\partial \varphi }{\partial \rho }\right)+\frac{1}{\rho^{2}}\frac{\partial^{2}\varphi }{\partial \phi^{2}}+\frac{\partial^{2}\varphi }{\partial z^{2}}=0$$

Although the Laplace equation of a constant current field is applicable to source-free regions and uniform linear medium, for an inhomogeneous medium, the equation must be partitioned.

#### 2.3.1. Boundary Conditions for Constant Current Fields

For a particular field, we can solve the potential function using equations from mathematical physics. A solution requires a discussion of the boundary value problem of the particular field. First, we derived the Laplace equation from the basic equations of the electric field. The potential function *φ* appears in the Laplace equation, which is solved based on the concrete boundary conditions. At the interface of two conducting mediums, the current density or electric field strength may be a discontinuous distribution because of the difference in conductivity of the two sides. The quantitative relation between the electric field and current on the interface can be calculated [18].

We constructed a cylinder on the interface, with a very small height and where the area of the circular base was ΔS. According to the current continuity equation on the cylinder,

(6) $$∯{}_{S}J\cdot dS=J_{1n}\Delta S-J_{2n}\Delta S=0$$

Then,

(7) $$J_{1n}=J_{2n}$$

On two sides of the interface of the two conducting mediums, the normal component of the current density is continuous.

We took a rectangular closed path on the interface, where the length is Δl and the width is very small. Conducting the line integral for electric field strength along this closed path,

(8) $$\oint {}_{l}E\cdot dl=E_{1t}\cdot \Delta l-E_{2t}\cdot \Delta l=\frac{J_{1t}}{\gamma_{1}}\cdot \Delta l-\frac{J_{2t}}{\gamma_{2}}\cdot \Delta l=0$$

Then,

(9) $$\frac{J_{1t}}{\gamma_{1}}=\frac{J_{2t}}{\gamma_{2}}$$

The tangential component of the electric field strength is continuous. From the above, we can deduce the boundary conditions of the constant current field on the adjacent conducting medium.

The boundary conditions of current density are given as:

(10) $$\left\{ \begin{array}{l} J_{1n}=J_{2n}\\ \frac{J_{1t}}{\gamma_{1}}=\frac{J_{2t}}{\gamma_{2}} \end{array}\right.$$

The boundary conditions of the electric field strength are given as:

(11) $$\left\{ \begin{array}{l} \gamma_{1}E_{1n}=\gamma_{2}E_{2n}\\ E_{1t}=E_{2t} \end{array}\right.$$

Then,

(12) $$\left\{ \begin{array}{l} E_{1t}=E_{2t}\\ J_{1n}=J_{2n} \end{array}\right.\Rightarrow \left\{ \begin{array}{l} \varphi_{1}=\varphi_{2}\\ \gamma_{1}\frac{\partial \varphi_{1}}{\partial n}=\gamma_{2}\frac{\partial \varphi_{2}}{\partial n} \end{array}\right.$$

#### 2.3.2. The Detection of the Coordinates of the Contact Position

**Figure 3:**
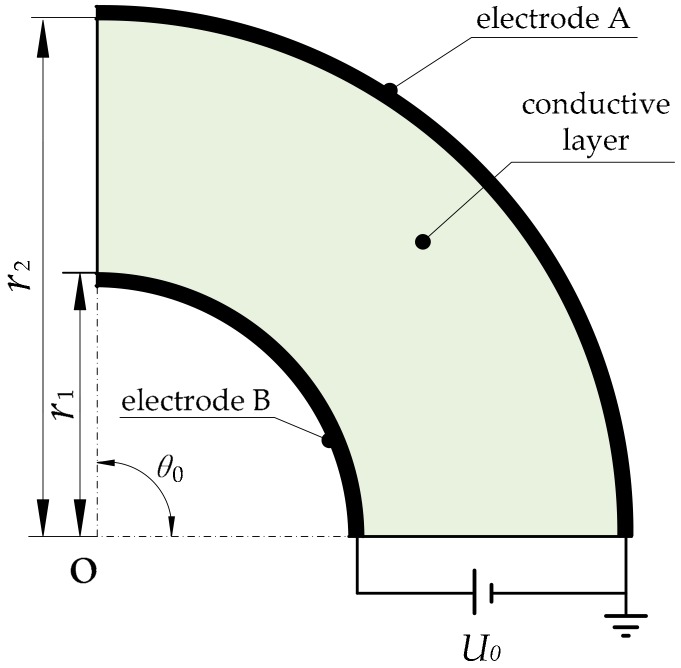
Method of constructing an electric field to detect the polar radius coordinate.

(1) Calculation of the polar radius:

We designed a pair of electrodes (A and B) on the outer and inner circle of the annular sector and applied a DC bias voltage across the electrodes, as shown in Figure 3.

Electrode A is connected with the ground and electrode B with the positive pole of the power supply. We list the boundary conditions,

(13) $$\left\{ \begin{array}{l} \begin{array}{l} \nabla^{2}\varphi =\frac{1}{\rho }\frac{\partial }{\partial \rho }\left(\rho \frac{\partial \varphi }{\partial \rho }\right)+\frac{1}{\rho^{2}}\frac{\partial^{2}\varphi }{\partial \phi^{2}}+\frac{\partial^{2}\varphi }{\partial z^{2}}=0\\ \varphi |{}_{r=r_{1}}=U_{0} \end{array}\\ \begin{array}{l} \varphi |{}_{r=r_{2}}=0 \end{array} \end{array}\right.$$

As the conductive layer is very thin, we ignore the influence in the z direction. Therefore, we have,

(14) $$\frac{\partial^{2}\varphi }{\partial z^{2}}=0$$

Furthermore, from the boundary conditions, we have:

(15) $$\frac{\partial^{2}\varphi }{\partial \phi^{2}}=0$$

Thus,

(16) $$\nabla^{2}\varphi =\frac{1}{\rho }\frac{\partial }{\partial \rho }\left(\rho \frac{\partial \varphi }{\partial \rho }\right)=0$$

and we obtain,

(17) $$\varphi =c_{1}\ln r+c_{2}$$

According to the boundary conditions, the constants of integration are determined to be:

(18) $$\left\{ \begin{array}{l} c_{1}=\frac{U_{0}}{\ln\frac{r_{1}}{r_{2}}}\\ c_{2}=-\frac{U_{0}\ln r_{2}}{\ln\frac{r_{1}}{r_{2}}} \end{array}\right.$$

Therefore, the voltage at the contact point is its potential minus the potential of electrode A (that is, the zero potential point).

(19) $$U_{r}=\varphi -0=\frac{U_{0}}{\ln\frac{r_{1}}{r_{2}}}\left(\ln r-\ln r_{2}\right)$$

**Figure 4:**
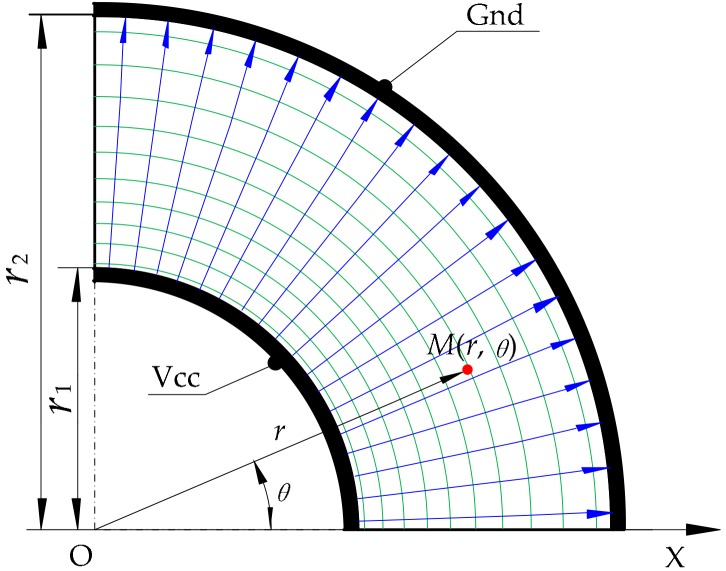
Diagram of the electric field distribution in the detection of the polar radius.

The formula for calculating the polar radius *r* is obtained as follows,

(20) $$r=r_{2}{\left(\frac{r_{1}}{r_{2}}\right)}^{\frac{{}^{U_{r}}}{U_{0}}}$$

where the radii of the inner circle $r_{1}$, outer circle $r_{2}$ and the voltage of power supply $U_{0}$ are all known. We just need to detect the potential $U_{r}$ of the corresponding point (that is, the contact point) to calculate the polar radius *r*. Hence, this constant electric field can be used to detect the polar radius *r* of the contact point. The electric field distribution of the conductive layer is shown in Figure 4.

**Figure 5:**
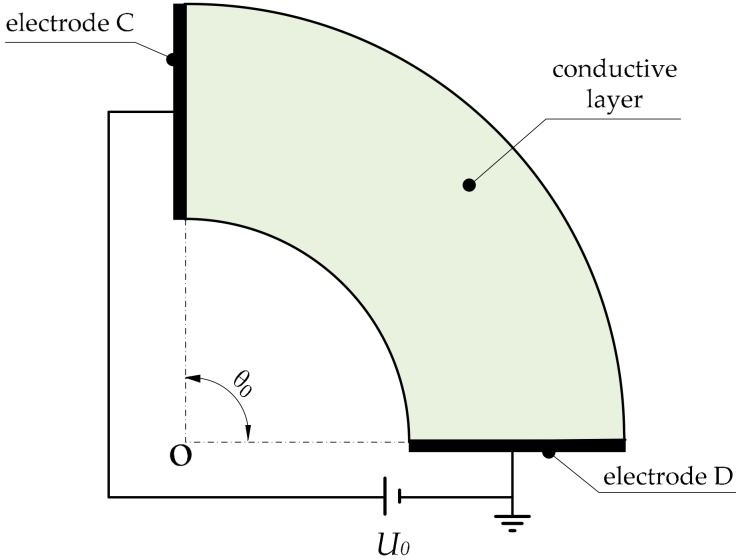
The setup for constructing an electric field to detect the coordinate of the polar angle.

(2) Calculation of the polar angle:

We set another pair of electrodes (C and D) on two linear edges of the other annular sector. We applied a DC bias voltage to the electrodes and established a polar coordinate system, as shown in Figure 5.

Electrode C is connected with the positive pole of the power supply and electrode D with the ground. In the polar coordinate system, let the potential φ be the field quantity to be solved. The boundary conditions of the conductive layer are,

(21) $$\left\{ \begin{array}{l} \nabla^{2}\varphi \left(\rho ,\theta ,z\right)=\frac{1}{\rho^{2}}\cdot \frac{\partial^{2}\varphi }{\partial \theta^{2}}=0\\ \varphi |{}_{\theta =0}=0\\ \varphi |{}_{\theta =\theta_{0}}=U_{0} \end{array}\right.$$

Integrating the Laplace equation, we obtain:

(22) $$U_{\theta }=C_{1}\theta +C_{2}$$

Then, the constants of integration are obtained from the boundary conditions,

(23) $$\left\{ \begin{array}{l} C_{1}=\frac{U_{0}}{\theta_{0}}\\ C_{2}=0 \end{array}\right.$$

**Figure 6:**
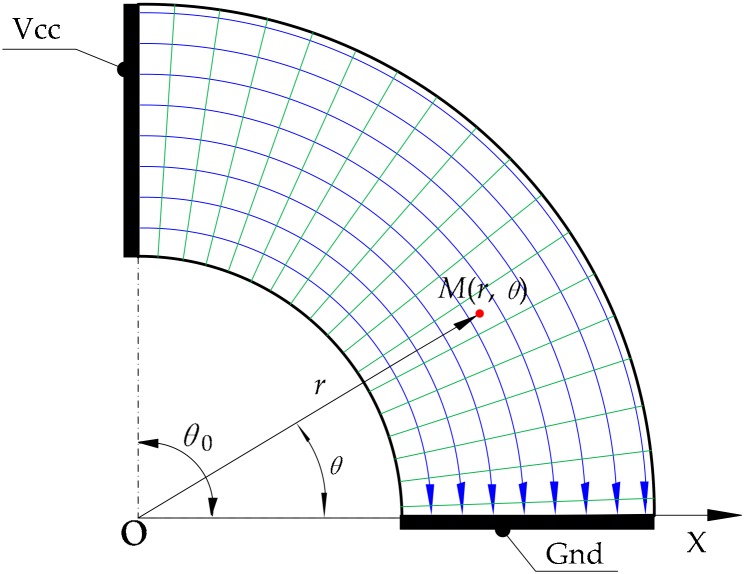
Diagram of electric field distribution in the detection of the polar angle.

Hence, the function between the detection value of potential $U_{\theta }$ and polar angle θ in the conductive layer can be obtained.

(24) $$\theta =\frac{U_{\theta }}{U_{0}}\theta_{0}$$

where the central angle $\theta_{0}$ of the annular sector and the voltage $U_{0}$ of the power supply are known. We just need to detect the potential $U_{\theta }$ of the corresponding point (that is, the contact point) to calculate the polar angle θ. The electric field distribution of the conductive layer is shown in Figure 6. The potential value decreases along the direction of the electric field lines.

### 2.4. A Three-Dimensional Sensor Model

**Figure 7:**
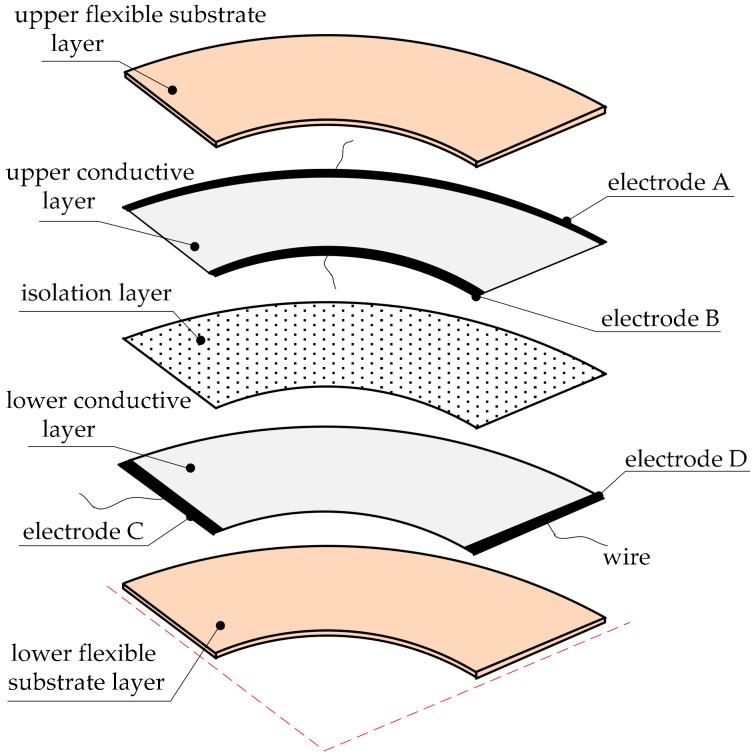
The structural model of the sensor.

This paper proposes a structure of the sensor as shown in Figure 7. There are five layers in this annular sectorial sensor. From top to bottom are the upper flexible substrate layer, upper conductive layer, isolation layer, lower conductive layer and lower flexible substrate layer. The flexible substrate layer can lead to a protective effect. We used a viscoelastic material to make this layer. The material has a certain thickness, which can delay the collision time when a collision occurs. It can also reduce the collision force and attenuate collision energy. The conductive layer is composed of a uniform conductive dielectric surface, electrodes and wires.

The first electric field is constructed on the upper conductive layer, and electrodes are arranged on the two circles of the annular sectorial conductive surface. The second electric field is constructed on the lower conductive layer, and electrodes are arranged on the two linear edges of the annular sectorial conductive surface. The electrode pair (B and A) of the upper conductive layer and the electrode pair (C and D) of the lower conductive layer were connected with the positive and negative poles of the DC power supply. A bias voltage was applied to each electrode pair to construct a constant current field on the conductive surface. The isolation layer insulates the upper conductive layer and lower conductive layer when there is no external force.

**Figure 8:**
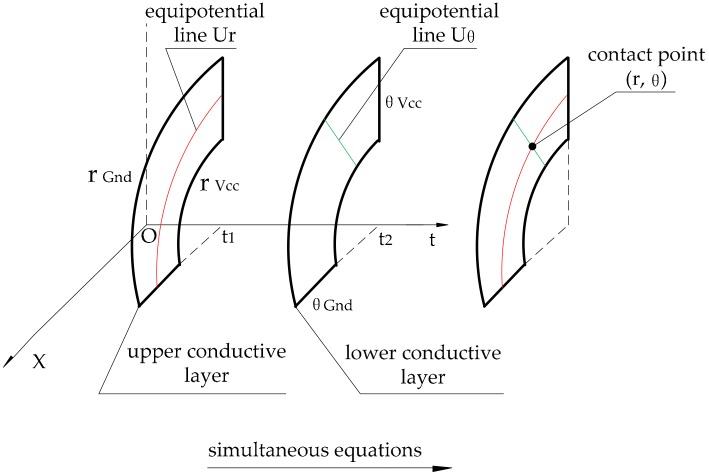
Principle of position detection.

According to the theoretical model given above, it is necessary to detect the potentials $U_{r}$ and $U_{\theta }$ to solve for the polar radius and the polar angle. For this purpose, one of the conductive layers should be the measuring layer when the other is constructing the electric field, as shown in Figure 8.

## 3. Simulation Analysis of the Sensor

The proposed sensor was simulated using COMSOL Multiphysics software (5.2.0.166, COMSOL, Inc., Stockholm, Sweden). A model of the upper and lower conductive layers was established, and the electric field distribution of the conductive layer was analyzed. The theoretical model of the electric field distribution was verified by simulation, and the performance of sensors under the point contact and surface contact in the simulation model was probed.

### 3.1. Simulation Result of Upper/Lower Conductive Layer

**Figure 9:**
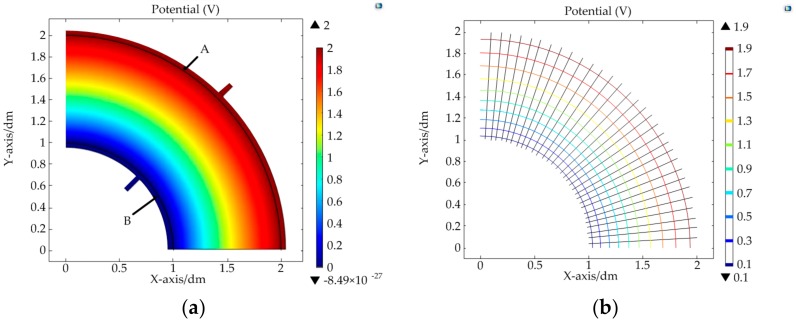
Simulation results of the upper conductive layer: (**a**) distribution map of the electric potential; (**b**) diagram of the electric field line and equipotential line.

**Figure 10:**
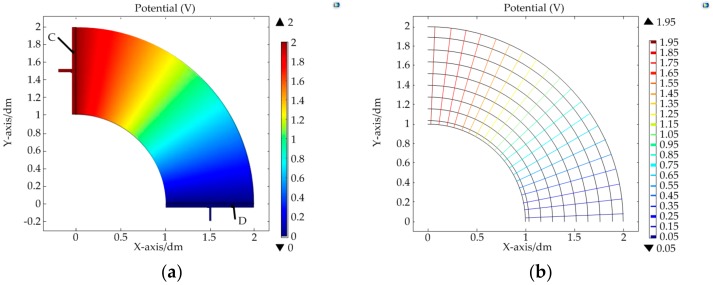
Simulation results of the lower conductive layer: (**a**) distribution map of the electric potential; (**b**) diagram of the electric field line and equipotential line.

The DC bias voltages applied on the upper/lower conductive layer were all $U_{0}=2V$. The simulation results are shown in Figure 9 and Figure 10.

The colored lines in Figure 9b and Figure 10b are the equipotential line and the black lines are the electric field lines. From the simulation results, it was found that a constant electric field could be constructed in the target region after meeting the constraint conditions of the electrodes. In the upper conductive layer, the electric potential was only related to the polar radius. In addition, for the same potential difference ΔU, when the polar radius r was larger, the corresponding increment Δr of the polar radius was also larger. That is to say, the sensitivity of the sensor is nonlinear in the direction of the polar radius. It can be calculated by differentiating $U_{r}$ in Equation (19):

(25) $$S_{n_{r}}=\frac{dU_{r}}{dr}=\frac{U_{0}}{r\left(\ln r_{1}-\ln r_{2}\right)}$$

In the lower conductive layer, the electric potential was only related to the polar angle. The equipotential lines on the conductive layer were arranged at equal angle intervals from electrodes C–D. It was linear in the direction of the polar angle. The sensitivity in this direction could be deduced according to Equation (22):

(26) $$S_{n_{\theta }}=\frac{\Delta U_{\theta }}{\Delta \theta }=\frac{U_{0}}{\theta_{0}}$$

### 3.2. Error Analysis of the Conductive Layers

#### 3.2.1. Contact Simulation of Upper Conductive Layer

**Figure 11:**
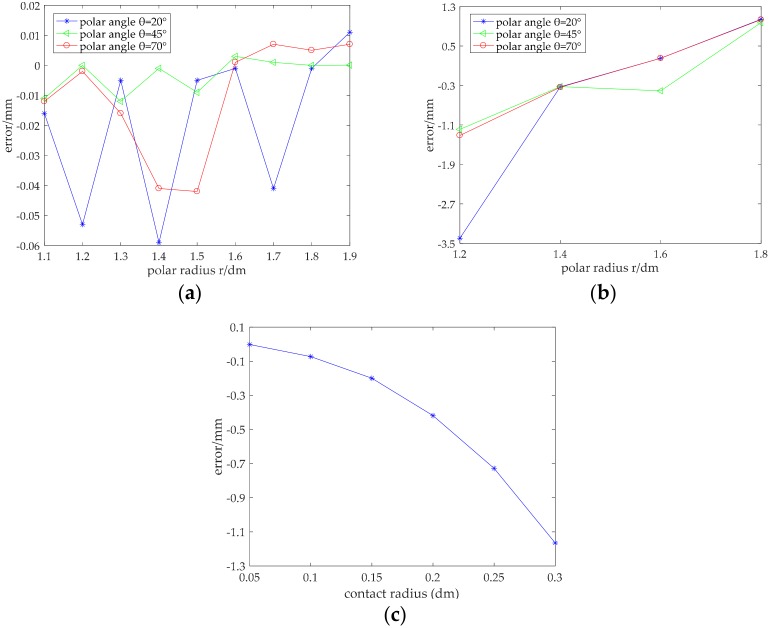
Detection precision of the polar radius: (**a**) error diagram of the polar radius under the point contact; (**b**) error diagram of the polar radius with surface contact; (**c**) influence of contact area on precision.

Error analysis was carried out using COMSOL Multiphysics software. Different contact conditions were simulated by adjusting the parameters of the contact area. First, the contact simulation of the upper conductive layer was carried out. We obtained the errors of the polar radius by changing the contact area and contact position, which is called the one-factor-at-a-time method, as shown in Figure 11.

Figure 11a,b show the error analysis of the polar radius in the case where the contact area is a circle whose radius is 1 mm and 15 mm, respectively. Figure 11c shows the influence of the contact area on precision, where the coordinate of the center point of the contact area was (150 mm, 45°). It can be seen from Figure 11a that the absolute error of the polar radius measured in the upper conductive layer was within 0.06 mm when the contact area was very small (that is, a point contact). It can be seen from Figure 11b that the measured absolute error of the polar radius was within 3.5 mm when the radius of the contact area was 15 mm. Although the error increased relative to a point contact, the error value was still sufficiently less than the contact radius, and so, the accuracy of detection was still high enough. Furthermore, as can be seen from both Figure 11a,b, when the contact area was unchanged, the error was larger around the edge of conductive layer, and the error was smaller around the center of the conductive layer. That is to say, the detection accuracy was higher around the center of the conductive layer. It can be seen from Figure 11c that the detection error of the polar radius increased when the radius of the contact area increased, if there were no other influencing factors. This is because strictly speaking, point contact is not possible—the contact area must have a certain area. The contact resistance in the contact area affects the detection accuracy. Moreover, because the mathematical model of the polar radius in this paper is a nonlinear equation, the error value of the coordinate measurement was affected by the rounding error of decimals. However, as most of the simulation values were smaller than the theoretical ones, we could modify the polar radius by adding an error compensation to decrease the detection error to some degree.

#### 3.2.2. Contact Simulation of the Lower Conductive Layer

**Figure 12:**
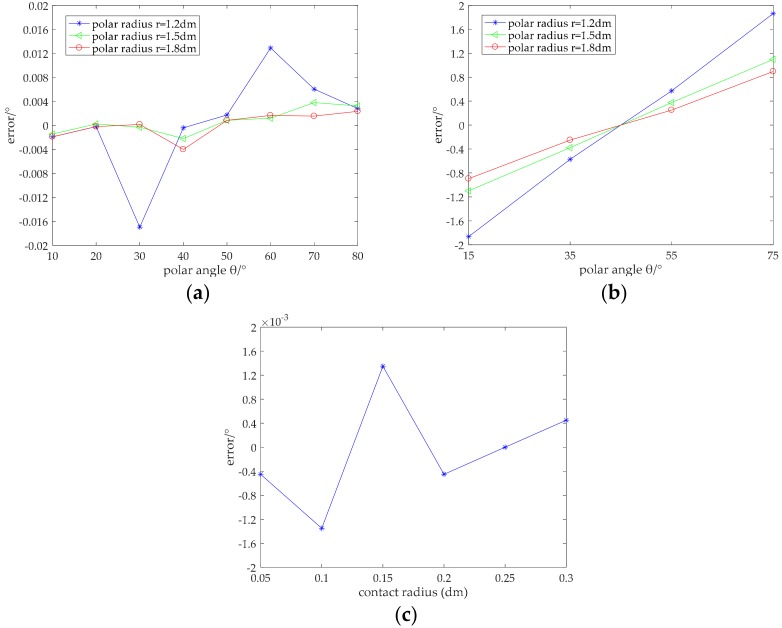
Detection precision for the polar angle: (**a**) error diagram of the polar angle under point contact; (**b**) error diagram of the polar angle under surface contact; (**c**) influence of the contact area on precision.

The constant electric field in the lower conductive layer is different from that of the upper conductive layer, manifested as the model of the upper conductive layer being a nonlinear equation, whereas the lower conductive layer has a linear equation. Therefore, it was necessary to conduct finite element analysis of the point contact and surface contact for the lower conductive layer. Similarly, the electrical conductivity of the lower conductive layer was the same as that of the upper conductive layer. We obtained error information of the polar angle by changing the contact area and contact position, as shown in Figure 12.

Figure 12a,b shows errors in measurement of the polar angle in the case where the contact area is a circle whose radius is 1 mm and 15 mm, respectively. Figure 12c shows the influence of the contact area on precision, where the coordinate of the center point of the contact area is (150 mm, 45°). It can be seen from Figure 12a that the absolute error of the polar angle measured in the lower conductive layer was within 0.018°, and the relative error was 0.06% when the contact area was very small (that is, for point contact); the detection accuracy was high. It can be seen from Figure 12b that the measured absolute error of the polar angle was within 1.9° and the relative error was 4% when the radius of contact area was 15 mm. Although the error was higher than for point contact, it did not exceed the corresponding angle range of the contact area. The detected polar angle still demonstrated a high detection accuracy. Comparing the polar angle errors under the same polar radius when the contact area was fixed, the errors were symmetrically distributed about θ=45°. This indicates that the error was small in the middle region of the conductive layer and became larger when close to the electrodes. This may be because the influence of the contact resistance was larger when closer to the electrode. As can be seen from Figure 12c, unlike in the upper conductive layer, the change of the contact area in the lower conductive layer had no significant effect on detection accuracy. The absolute error was always within 0.0014°, and the relative error was 0.0031%, meaning a high degree of accuracy.

In summary, the detection accuracy was affected by the contact area and was higher in the middle region than at the edge, regardless of whether it was on the upper conductive layer or lower conductive layer. The closer to the edge, the greater the error was.

## 4. Experiment

### 4.1. Fabrication and Wrapping Test of Sensor Samples

**Figure 13:**
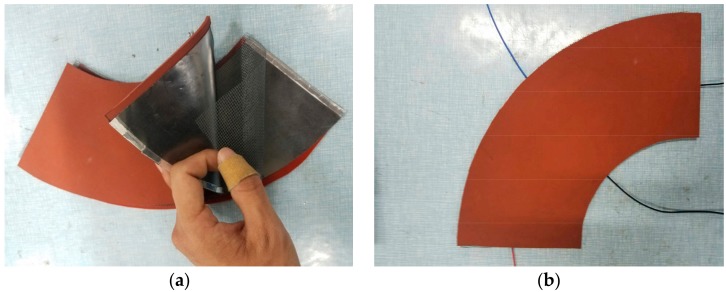
Sensor sample: (**a**) before packaging; (**b**) after packaging.

The link of a FARO Gage (a kind of coordinate measuring machine, FARO Technologies, Inc., Lake Mary, FL, USA) was used as the target structure to test the sensor. A range of materials could satisfy the corresponding characteristics, with the main requirement being flexibility. The substrate required a certain thickness to protect the sensor. The conductivity of the electrode was far greater than that of conductive layer, in order to create a constant electric field. Otherwise, the mathematical model of the electric field was not identical to that analyzed above. The isolation layer must be neither too thick nor too thin, because it would be hard to detect if this layer were too thick when the collision occurred, and a malfunction will occur if the layer is too thin even when there is no collision. The materials were selected according to these requirements. Graphite paper was selected as the material of the conductive layer, aluminum foil for the electrode, polyethylene mesh as the isolation layer, silicone foam sheet as the flexible substrate layer and conductive adhesive tape as the auxiliary material. The sensor sample was fabricated to match the geometric dimensions of the link of the FARO Gage, based on the structure model shown in Figure 7. The fabricated sensor sample is shown in Figure 13.

Figure 13a shows the different layers of the sensor before packaging. The upper conductive layer had been bonded to the upper flexible substrate layer, and the lower conductive layer had also been bonded to the lower flexible substrate layer. Figure 13b shows the sensor sample after packaging.

**Figure 14:**
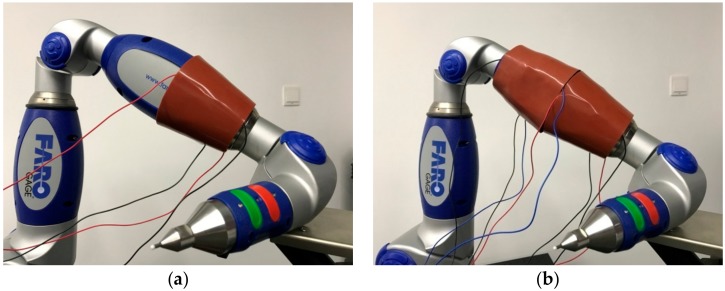
Sensor wrapping with: (**a**) one sensor; (**b**) two sensors.

It can be seen from the structure of the FARO Gage that the target link had the shape of a rugby ball, which can be regarded as a connection of two truncated cones. Therefore, two annular sectorial sensors are required to wrap the link. The results of wrapping the sensor around the link are shown in Figure 14.

The annular sectorial sensor, which can be seen from Figure 14, has yet to fully realize seamless wrapping, but the advantage is still obvious when compared with a common rectangular sensor wrapped around an irregular link.

### 4.2. Experiment of Sample Contact

**Figure 15:**
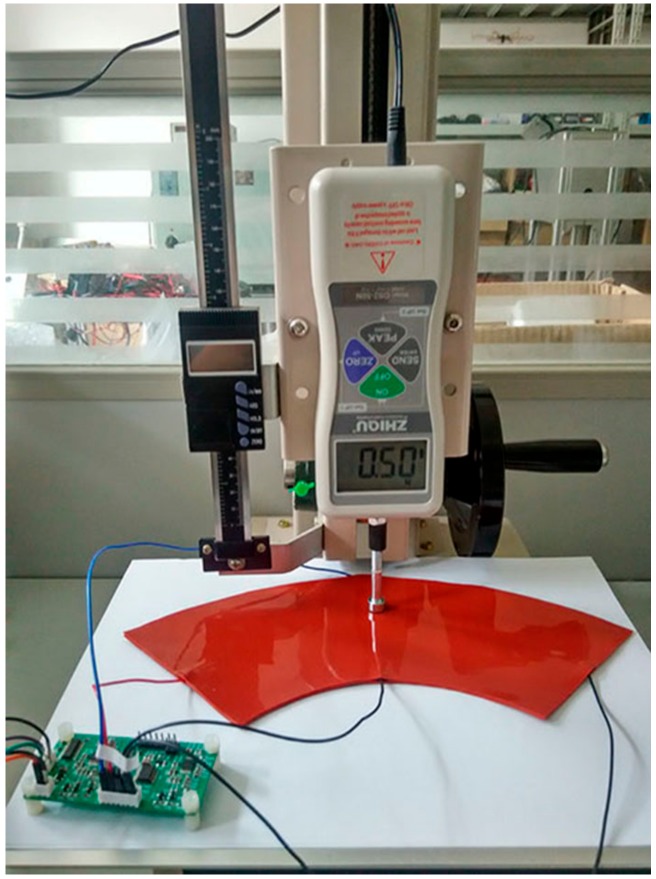
Setup of the contact experiment.

**Figure 16:**
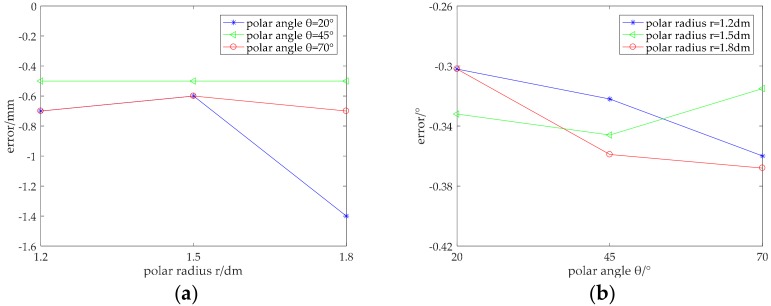
Experimental errors for a point contact: (**a**) error in polar radius; (**b**) error in polar angle.

**Figure 17:**
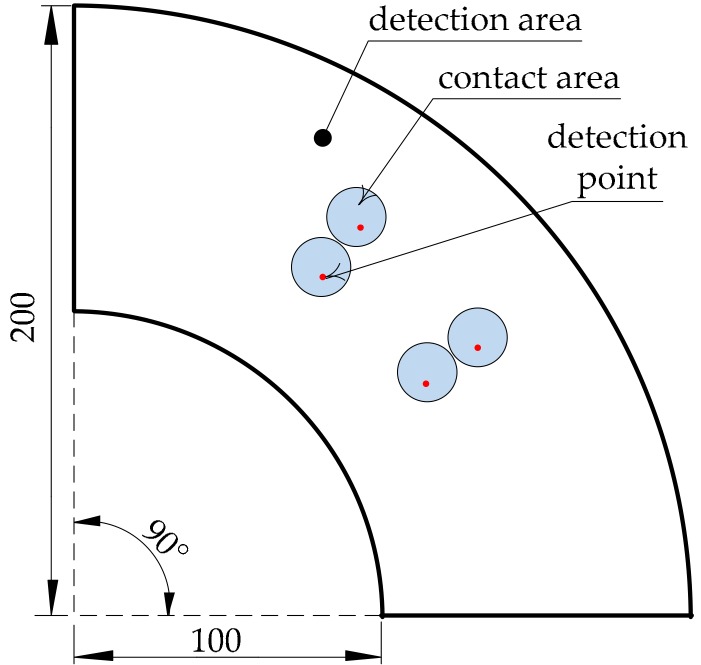
Comparative diagram between the actual contact area and detected points.

We used the packaged sample as shown in Figure 13b to carry out the experiment. A force gauge was used to test the precision of the sensor sample. The sample was then placed on the force gauge, as shown in Figure 15. We alternately applied a DC voltage of +1 V to the two pairs of electrodes described above, using time-division multiplexing through the control circuit. This meant that the two conductive layers experienced electric fields at different times. When one conductive layer produces a constant electric field, the other could be used as the guide layer to measure the potential of the contact point. We operated the rocker to press the required point on the sample, and when it reached the threshold force (the force that enables the two conductive layers to make contact with each other, i.e., 0.5 N in this case), the guide layer could measure the potential of the contact point. Next, the coordinate on the former conductive layer was deduced according to the mathematical model. The one-factor-at-a-time method was also used in the experiment of the sample contact to explore the effect of different position and contact area on measurement error. The experimental results of the sample contact are shown in Figure 16 and Figure 17.

Figure 16 shows the error analysis of the position detection in the case of a small contact area (point contact). It can be seen from Figure 16a that the absolute error of the polar radius of the sample was within 1.6 mm, which was much larger than the 0.06 mm obtained in the simulation. However, the relative error was still within 1%, which indicates that the accuracy of the detection of the sample was high. Similarly, the absolute error of the polar angle was within 0.5°. The experiment was conducted repeatedly, and the results did not change. Therefore, the repeatability and stability are reliable.

Table 2 shows the position coordinates and errors measured in the case where the contact radius was 9.5 mm, i.e., a surface contact. It can be seen from the table that the experimental error of the surface contact was larger than that of the point contact, because the expansion of the contact area had an impact on the detection accuracy. The experiment error of surface contact was larger than that in the simulation. In human-robot interaction, especially human-robot collision, more attention is paid to whether the detected coordinates fall within the contact area. It can be seen from Figure 17 that the points measured by the sensor sample fell within the contact area. Therefore, the detection accuracy of the sensor sample can still meet practical requirements.

Compared to the simulated data, the position error of the sample was larger, but still within the acceptable error range. The reasons for this increase may be as follows. (1) The materials of the conductive layer were not uniform enough. Materials can be customized later to improve accuracy. (2) Sensor samples were made by hand, so the process was relatively crude, and the cutting of the geometric shape showed deviation. (3) Suturing of the aluminum foil and conductive layer was not good, which added additional impedance to the conductive layer, resulting in a low potential value. In future work, the detection accuracy of the sensor can be improved by improving the materials used in the sensor, improving the process accuracy and adding error compensation to the mathematical model.

## 5. Conclusions

This paper reports a flexible annular sectorial sensor that can be wrapped on a truncated cone-shaped link and can continuously measure the coordinates of a contact position. We started from electric field theory and established a mathematical model combining the electric potential and position coordinate based on the boundary conditions. Furthermore, finite element analysis and the theoretical error analysis were carried out. A physical sensor sample was successfully developed and tested. The maximum errors of the sensor sample did not exceed 1.6 mm and 0.5° in the polar radius direction and polar angle direction, respectively, using a point contact, and the detected coordinates fell within the contact area for a surface contact. The sensor could be effectively wrapped on a truncated cone-shaped link. The experimental results showed that the sensor designed in this paper is feasible, providing a possible solution for robot contact and collision detection.

## Figures and Tables

**Table 1:** Characteristics of tactile sensors.

Characteristic	Array Sensor	Regular Non-Array Sensor
adaptability	grasp sensing	larger surface sensing
continuity	discontinuous detection	continuous detection
simplification	complex circuits	simple circuits

**Table 2:** Experiment results for surface contact.

Time	Theoretical Value	Actual Value	Error
1	(140 mm, 35°)	(137.5 mm, 33.86°)	(−2.5 mm, −1.137°)
2	(140 mm, 55°)	(137.7 mm, 54.06°)	(−2.3 mm, −0.937°)
3	(160 mm, 35°)	(158.1 mm, 34.02°)	(−1.9 mm, −0.985°)
4	(160 mm, 55°)	(157.9 mm, 53.90°)	(−2.1 mm, −1.09°)
